# Genome and metabolome mining of marine obligate Salinisporsatrains to discover new natural products

**DOI:** 10.3906/biy-1807-136

**Published:** 2019-02-07

**Authors:** Süleyman ÖZAKIN, Ebru İNCE

**Affiliations:** 1 Center for Marine Biotechnology and Biomedicine, Scripps Institution of Oceanography, University of California San Diego , La Jolla, California , USA; 2 Department of Biology, Faculty of Science, Dicle University , Diyarbakır , Turkey

**Keywords:** Salinispora, genome mining, metabolomics, mass spectrometry, GNPS, marine natural products

## Abstract

Marine microorganisms are receiving more attention as a promising potential source of new natural products. In the present study, we performed genomic and metabolomic analyses to explore the metabolic potential of the obligate marine actinomycete genus *Salinispora*. The genomes of thirty *Salinispora* strains were prospected in search of biosynthetic gene clusters including polyketide synthase (PKS), nonribosomal peptide synthetase (NPRS), terpene, indole, lantibiotics, and siderophores. We determined considerable diversity of natural product biosynthetic gene clusters in their genome. There were a total of 1428 putative gene clusters involved in the biosynthesis of various bioactive natural products. Furthermore, 1509 ketosynthase (KS) and condensation (C) domains were detected by using NapDoS belonging to PKS and NRPS genes, respectively. Metabolic profiling was performed by a nontargeted LC-MS/MS approach combined with spectral networking using Global Natural Product Social Molecular Networking (GNPS). Dereplication and tentative identification of natural products were evaluated for common chemical properties and their associated pathways. Significant bioactive natural products such as lomaiviticin C, 7-OH-staurosporine, staurosporine, and cyanosporaside B were determined. More importantly, an unknown glycosylated compound associated with an NRPS/PKS-I hybrid gene cluster in *Salinispora pacifica* CNY703 was established through chemical and genomic analyses.

## 1. Introduction

Natural products are biological molecules produced by organisms such as fungi, plants, and microorganisms. The biotechnological potential of natural products from microorganisms is receiving more attention for the discovery of novel bioactive compounds (Qin et al., 2017). Of all microbes, the actinomycetes traditionally represent one of the most important sources. Marine actinomycetes belonging to the genus *Salinispora* have long been an important source of structurally diverse and biologically active natural products, several of which have inspired the development of new classes of therapeutic agents (Feling et al., 2003; Fenical and Jensen, 2006; Jensen and Mafnas, 2006). Polyketide- and peptide-derived metabolites are among the most diverse and include many clinically important compounds (Fischbach and Walsh, 2006; Jang et al., 2013).

In recent years, genomics and metabolomics have been combined to identify new bioactive metabolites. The mining of actinomycete genomes has proven to be useful in the identification of secondary metabolite biosynthetic gene clusters (Jensen and Mafnas, 2006; Zerikly and Challis, 2009). In untargeted metabolomic studies, liquid chromatography followed by mass spectrometry (LC/MS) has been widely used to detect the highest number of metabolites in small amounts of sample. Identification of these compounds is based on tandem MS (MS/MS) data, produced by fragmenting the compound and determining the masses of the fragments. Global Natural Products Social Molecular Networking (GNPS, http://gnps.ucsd.edu) is an open-access knowledge base for public sharing of processed and annotated MS/MS spectrometry data. The molecular networks created by GNPS enable dereplication (rapid identification of known metabolites) and structural identification of metabolites through spectrum library matching. The MS-guided genome mining technique helps to bridge the gaps between genes, pathways, and chemical features of metabolites. It creates an algorithm capable of comparing characteristic fragmentation patterns, thus composing molecular groups with the same structural features and probably the same biosynthetic origin (Wang et al., 2016). In this respect, we used a combined genomic and metabolomic mining approach to highlight the natural product biosynthetic capacity of 30 marine obligate *Salinispora* strains.

## 2. Materials and methods

### 2.1. Bacteria and fermentation studies

The names of the 30 *Salinispora* strains used in this study and their genome accession numbers are listed in Table 1. Glycerol stock solutions of all bacteria were prepared by inoculating 10 μL of cell stock into 25 mL of A1 medium containing 10 g/L starch, 4 g/L yeast extract, 2 g/L peptone, and 22 g/L Instant Ocean sea salt (Instant Ocean®) at pH 6.5 and were incubated at 25 °C for 6–10 days.

**Table 1:** *Salinispora* strains used in this study and genome accession numbers.

Strains	Genome accession numbers
*Salinispora pacifica* CNS801	2561511036
*Salinispora pacifica* CNY703	2563366517
*Salinispora pacifica* CNS860	2518285563
*Salinispora pacifica* CNR909	2561511038
*Salinispora pacifica* CNY666	2563366532
*Salinispora pacifica* CNY239	2524614561
*Salinispora pacifica* CNT796	2515154182
*Salinispora pacifica* CNT603	2515154185
*Salinispora pacifica* CNT124	2517572159
*Salinispora pacifica* CNQ768	2517572155
*Salinispora pacifica* CNT851	2517572162
*Salinispora arenicola* CNR107	2519103194
*Salinispora arenicola* CNY011	2517572153
*Salinispora arenicola* CNY230	2561511115
*Salinispora arenicola* CNR425	2528311033
*Salinispora arenicola* CNY256	2518285559
*Salinispora arenicola* CNS820	2565956528
*Salinispora arenicola* CNS299	2524614529
*Salinispora arenicola* CNT800	2515154088
*Salinispora arenicola* CNS673	2519103185
*Salinispora arenicola* CNH877	2519103192
*Salinispora arenicola* CNH963	2524023246
*Salinispora arenicola* CNY679	2561511113
*Salinispora arenicola* CNS325	2571042009
*Salinispora arenicola* CNT798	2515154186
*Salinispora arenicola* CNH643	2561511037
*Salinispora arenicola* CNT850	2515154135
*Salinispora arenicola* CNH962	2519103193
*Salinispora arenicola* CNT799	2526164509
*Salinispora tropica* CNT250	2540341193

For the production of metabolites, all isolates were grown in triplicate in 100 mL of A1M1 medium containing 5 g/L starch, 2 g/L yeast extract, 2 g/L peptone, and 22 g/L Instant Ocean sea salt at pH 6.5 and were incubated at 25 °C with shaking at 160 rpm for 14–20 days.

### 2.2. Genome mining

A total of 30 *Salinispora* genomes were downloaded from the Joint Genome Institute's Integrated Microbial Genomes (IMG) database (http://img.jgi.doe.gov). The draft genome sequences of all *Salinispora* strains were analyzed by NapDoS (Ziemert et al., 2012) and antiSMASH 2.0 (Blin et al., 2013). NapDoS was used to detect and extract KS and C domains in PKS and NRPS genes in the genomes, respectively. antiSMASH 2.0 was used to detect secondary metabolite biosynthetic gene clusters with the whole range of known secondary metabolite compound classes, including polyketides, nonribosomal peptides, lantipeptides, oligosaccharide antibiotics, phenazines, thiopeptides, homo-serine lactones, phosphonates, and furans. Homologous clusters for predicted biosynthetic pathways were analyzed using the MultiGeneBLAST program (Medema et al., 2013). DoBISCUIT software was also used to screen a variety of gene clusters for secondary metabolite biosynthesis (Ichikawa et al., 2012).

### 2.3. Extraction and spectroscopic analysis of metabolites

The supernatant and pellets were extracted with ethyl acetate (1:1, v/v). The extracts were dried with Na_2_SO_4_ and evaporated to dryness under reduced pressure to yield crude extracts. After being weighed, the extracts were dissolved in methanol to obtain a final concentration of 1 mg/mL. LC and LC-MS/MS analyses were carried out after filtration through 0.2-μm Acrodisc MS syringe filters (25 mm).

The samples were injected as 20 μL into an Agilent 1260 LC system with an Agilent Extend-C18 RP UPLC column (2.1 × 100 mm, 1.8 μm) connected to an Agilent 6530 Accurate-Mass Q-TOF LC/MS. The LC gradient was as follows: 10% (v/v) acetonitrile (ACN) (0.1% water, 0–3 min), 10–100% (v/v) ACN (0.1% water)/0.1% water (3–23 min), 100% ACN (0.1% water, 23–25 min), 10% (v/v ACN (0.1% water, 25–30 min). The column compartment temperature was 25 °C.

Q-TOF MS settings during the LC gradient were as follows: acquisition mass range m/z 100–1600, MS scan rate 1s^-1^, MS/MS scan rate 2s^-1^, fixed collision energy 20 eV, source-gas temperature 300 °C, gas flow 11 L min^-1^, nebulizer 45 psi, ion polarity positive; scan source parameters—VCap 3000, Fragmentor 100, Skimmer1 65, OctopoleRFPeak 750. The MS was autotuned using Agilent tuning solution in positive mode before each measurement. LC (DAD) and MS data were analyzed with ChemStation and MassHunter software (Agilent), respectively.

### 2.4. GNPS molecular networking and dereplication analyses

The raw MS/MS data of 30 *Salinispora* strains were converted from MassHunter data files (d.) to mzXML file format using the Trans Proteomic Pipeline.

MS/MS data of molecular network and dereplication analyses were determined with GNPS molecular networking. Before the analyses, spectral networks were imported into Cytoscape 3.1.0 for visualization as a network. Cytoscape software was employed to visualize the molecular networks and biological pathways. It integrates these networks with annotations, gene expression profiles, and other state data (www.cytoscape.org). Some parent ions obtained from LC/MS data were analyzed with METLIN (Smith et al., 2005) and Marinlit (http://pubs.rsc.org/marinlit/) databases. Glycogenomic analyses were carried out as described previously (Kersten et al., 2013).

## 3. Results and discussion

### 3.1. Genomic analysis of secondary metabolite genes

**Figure 1:**
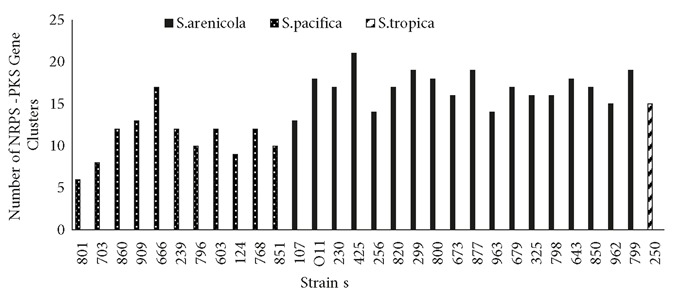
The number of NRPS-PKS pathways of Salinispora strains detected by antiSMASH.

Based on the genome analysis, *Salinispora* strains appeared to be rich sources for production of various chemical entities and new secondary metabolites. They have a large proportion of natural product biosynthetic gene clusters (about 9.9%) in their genomes in comparison to terrestrial counterparts *Streptomyces coelicolor* (4.5%) and *Streptomyces avermitilis* (6%) (Bentley et al., 2002; Ikeda et al., 2003). According to antiSMASH results, 1428 putative natural product gene clusters were found to be involved in the biosynthesis of various pathways such as NRPS, PKS, terpenes, butyrolactones, siderophores, bacteriocins, and lantibiotics. In total, 1509 ketosynthase (KS) and condensation (C) domains were detected in all strains belonging to their PKS and NRPS genes by using NapDoS, respectively. NRPS and PKS gene clusters were remarkably higher than other biosynthetic genes in almost all strains (Table 2). The *Salinispora arenicola* strains, particularly CNR425, were identified as hot spots for PKS and NRPS biosynthetic capacity (Figure 1). Among the 30 *Salinispora* strains, *S. arenicola* CNS673 and *S. arenicola* CNT799 both contain eleven PKS-I pathways. In addition, the highest number of PKS-II biosynthetic gene clusters was identified in both *S. pacifica* CNT851 and CNT796 strains. It has been previously reported that certain *Salinispora* genomes were enriched in PKS and NRPS biosynthetic pathways (Ziemert et al., 2014). Furthermore, the average number of natural product gene clusters identified per genome was significantly greater in *S. arenicola* than in *S. pacifica* and *S. tropica* (Letzel et al., 2017). In our study, all *S. arenicola* strains produced the most popular polyketide compound, rifamycin, many of whose analogs have long been used to treat mycobacterial infections. Apart from *S. pacifica* CNS860, the other 29 strains host lymphostin biosynthetic gene clusters in their genomes. Highly conserved lymphostin gene clusters were also reported to exist in *Salinispora* bacteria by Miyanaga et al. (2011). *S. pacifica* CNS860 and *S. tropica* CNT250 strains have been able to produce salinosporamide A, which is currently being employed in clinical trials for the treatment of cancer (Feling et al., 2003). Sporolide biosynthetic gene clusters were determined in the genomes of two strains: *S. tropica* CNT250 and *S. arenicola* CN963. Cyanosporaside, salinilactam, and cyclomarin pathways were rarely seen in the analyzed strains. A total of 23 *Salinispora* strains could produce siderophores. These biologically active iron chelators have been playing important roles in adaption to unstable environmental conditions.

**Table 2:**
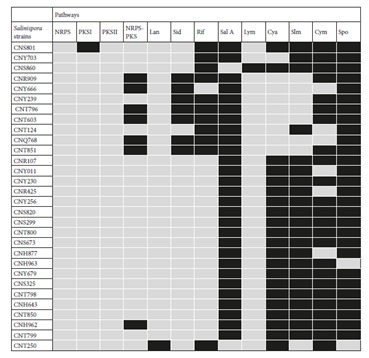
The pathways detected by AntiSMASH and NapDOS for *Salinispora* strains. The presence and absence of pathways are shown by filling the boxes with gray and black colors, respectively.

In the present study, it is observed that *S. arenicola* strains (CNR107, CNS673, CNS325, CNY230, CNR425, CNS820, CNH962, CNH963, and CNY299) encode biosynthetic genes related to glycosylated pathways in NPRS and PKS gene clusters. Three different putative glycosylated pathways were detected in *S. pacifica* CNY703 by the antiSMASH program. These pathways lead to the synthesizing of the following compounds: (a) staurosporine, (b) an unknown hybrid NRPS-PKS, and (c) a NRPS. The gene clusters of the first two pathways (staurosporine and an unknown hybrid NRPS-PKS) have common specific sugar biosynthetic genes, including NDP sugar epimerases, aminotransferase, 4,6-dehydratase, Glu1P N transferase, CH_3_ transferase, glycosyltransferase, and NDP hexose 2,3-dehydratase. These genes are important markers for the discovery of glycosylated natural products by glycogenomic approaches (Kersten et al., 2013). However, O-CH_3_ transferase, which is the specific glycosylation gene, was discovered only in the gene cluster of the unknown NRPS-PKS compound (b), and not in the staurosporine (a). It has been determined that the staurosporine and unknown NRPS/PKS-I gene clusters are homologs, but their products were synthesized by different pathways when analyzed by MultiGeneBlast.

### 3.2. Dereplication and molecular networking analysis by GNPS

Dereplication is an important approach to rapidly identify known natural products in complex extracts. To create a molecular network for dereplication and structural identification of metabolites, the GNPS database was used. Molecular networking is a platform that provides a summary of mass spectrometry-based metabolomics by comparing molecular properties with fragmentation patterns to highlight chemical relationships. Each node represents a consensus spectrum and edges represent related fragmentation patterns. Thus, clusters in the network are represented by the molecular ions that emphasize the structural relationships and similarities of the molecules.

**Figure 2:**
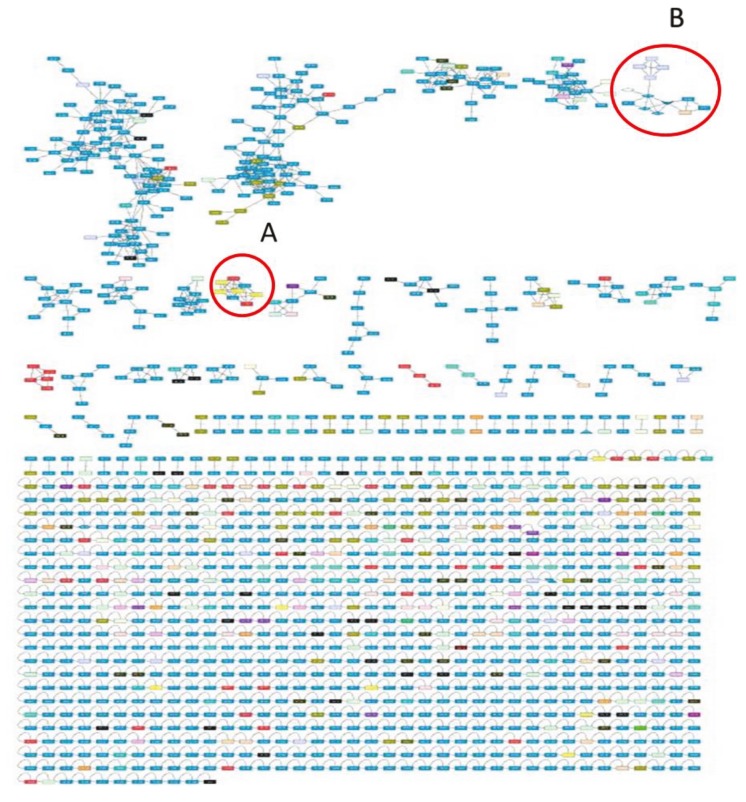
Molecular networking analysis of 30 strains of Salinispora. Clusters A and B represent the lomaiviticin and staurosporine clusters, respectively.

**Figure 3:**
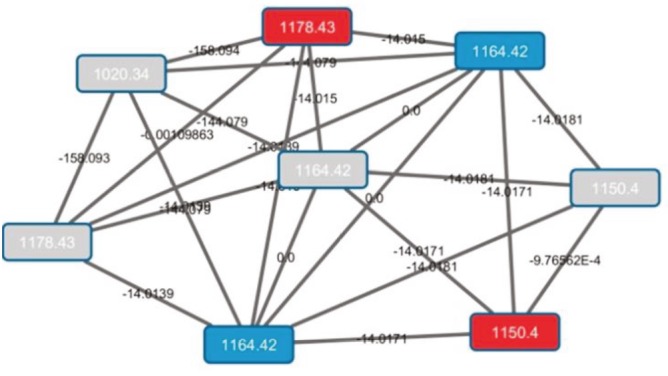
Cluster A (lomaiviticin) in networking.

Thirty strains were grown in triplicate and 90 bacterial crude extracts were obtained to analyze the metabolites with LC-MS/MS and subsequently with GNPS molecular networking. According to dereplication analysis, 21 strains were determined to produce some well-known compounds in the GNPS database (Table 3). A marine sponge-derived natural product, mycalamide A, which is known as a protein synthesis inhibitor with potent antitumor activity, was detected in two *S. pacifica* strains (CNY239 and CNT124). The isolation and in vitro antiviral activity of mycalamide A and mycalamide B were reported from a New Zealand sponge, *Mycale* (Perry et al., 1990). Lomaiviticin C is another bioactive natural product established in dereplication analysis. This antitumor compound was determined to be produced by two *S. pacifica* (CNT796, CNT603) and one *S. tropica* strain (CNT250). Salinisporazine A and enterocin were found in *S. arenicola* CNR425 and *S. pacifica* CNT796 extracts, respectively. The well-known antibiotic rifamycin was dereplicated from most of the *S. arenicola* strains. The indolocarbazole compound staurosporine, which has protein kinase inhibitory activity, is also a common compound in the bacterial crude extracts of 18 strains. After generating the molecular networks, node connectivity was visualized (Figure 2). One node (colored box) represents one consensus MS/MS spectrum that comes from the source files of the LC-MS/MS, which is labeled with the parent (precursor) mass. Furthermore, 1347 nodes and 1627 edges (linker of nodes) were included in the network, some of which are unique. The network also contained at least 40 different clusters (Figure 2). The black nodes show a unique spectrum relative to the A1M1 medium as a negative control. Lomaiviticin C, 7-OH-staurosporine, staurosporine, and cyanosporaside B clusters were identified in the molecular networking of the *Salinispora* metabolome. Strains *S. tropica* CNT250, *S. pacifica* CNT796, and *S. pacifica* CNT603 all produced an anticancer compound, lomaiviticin. Cluster A in the network was identified as lomaiviticin, which consists of the nodes from a collection of *S. tropica* CNT250, *S. pacifica* CNT796, and *S. pacifica* CNT603 (Figure 3). Red and gray nodes represent unique MS/MS spectra of *S. tropica* CNT250 and *S. pacifica* CNT796, respectively, while two blue nodes belong to each of the three strains. Glycogenomic analysis further proved that lomaiviticin C is a glycosylated compound containing oleandrose and pyrrolosamine as sugar moiety.

**Table 3:** The results of dereplication analysis of *Salinispora* strains.

Salinispora strains	GNPS library hits				
*S. pacifica* CNS801	-
*S. pacifica* CNY703	-
*S. pacifica* CNS860	-
*S. pacifica* CNR909	Cyanosporaside B
*S. pacifica* CNY666	-
*S. pacifica* CNY239	Cyanosporaside B	Mycalamide A
*S. pacifica* CNT796	Enterocin	Lomaiviticin C
*S. pacifica* CNT603	Cyanosporaside B	Lomaiviticin C
*S. pacifica* CNT124	Mycalamide A
*S. pacifica* CNQ768	-
*S. pacifica* CNT851	-
*S. arenicola* CNR107	7-OH Staurosporine	Staurosporine	Rifamycin S
*S. arenicola* CNY011	-
*S. arenicola* CNY230	7-OH Staurosporine	Staurosporine
*S. arenicola* CNR425	Salinisporazine A
*S. arenicola* CNY256	7-OH Staurosporine	Staurosporine	Rifamycin S	Saliniketal A	Aphidicolin
*S. arenicola* CNS820	7-OH Staurosporine	Staurosporine	Rifamycin S	Saliniketal A
*S. arenicola* CNS299	7-OH Staurosporine	Staurosporine	Rifamycin S	Rifamycin W
*S. arenicola* CNT800	Staurosporine
*S. arenicola* CNS673	7-OH Staurosporine	Staurosporine	Rifamycin S	Saliniketal A	Aphidicolin
*S. arenicola* CNH877	7-OH Staurosporine	Staurosporine	Rifamycin S	Saliniketal B
*S. arenicola* CNH963	-
*S. arenicola* CNY679	Staurosporine	Rifamycin S	Saliniketal A	Saliniketal B
*S. arenicola* CNS325	Rifamycin S	Saliniketal B
*S. arenicola* CNT798	Staurosporine	Saliniketal A	Saliniketal B
*S. arenicola* CNH643	Staurosporine	Rifamycin S
*S. arenicola* CNT850	Staurosporine	Rifamycin S
*S. arenicola* CNH962	-
*S. arenicola* CNT799	Staurosporine	Rifamycin S	Dinactin
*S. tropica* CNT250	Lomaiviticin C

**Figure 4:**
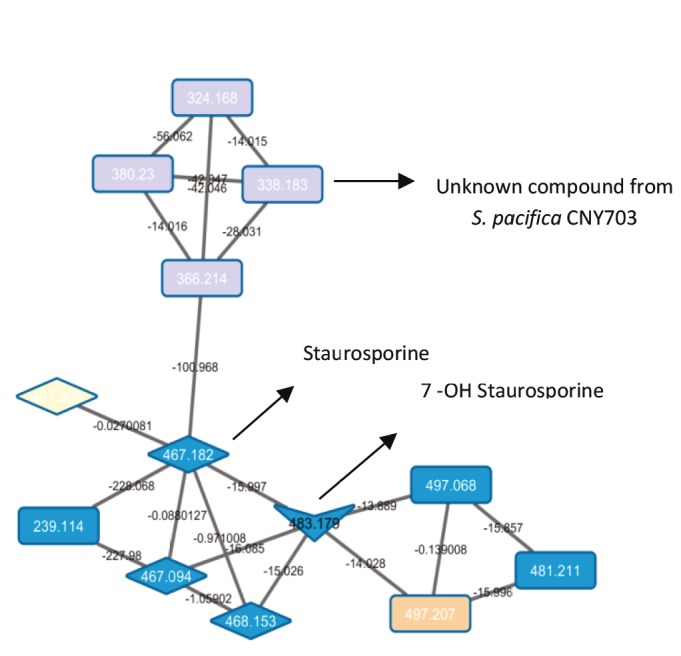
Cluster B in networking.

Cluster B represents an example of the identification of 7-OH-staurosporine by using MS-guided genome mining (Figure 4). In addition, there was a quartet constituted of nodes with masses m/z 324.168, 338.183, 366.214, and 380.23 in this cluster. These nodes (pale purple) come only from *S. pacifica* CNY703. Node m/z 366.214 in the quartet is connected to the staurosporine node (m/z 467.182). Although *S. pacifica* CNY703 has an indolocarbazole biosynthetic gene cluster in its genome, the production of staurosporine has not been detected in this strain. When examining the MS/MS fragmentation behavior of *S. pacifica* CNY703 for cluster B, it seems that the m/z 338.183, 366.214, and 380.23 signals are not parent but fragmented ions. The LC/MS chromatogram was investigated in the range of m/z 600–700. Two parent ions, m/z 661 [M+H] and 683 [M+Na], were identified in the chromatogram connected to fragmented ion m/z 366.214. Furthermore, the UV library search of the two related ions has a top hit matched with an unknown NRPS/PKS-I compound, CNS205. The molecular weight of the related compound was in the range of 650–700, which may correspond to glycine, tryptophan, valine/leucine, ketide chains, and sugar. These chemical features have been found in the core structure of the unknown NRPS/PKS-I hybrid compound according to monomer prediction by antiSMASH.

In a previous study, high levels of pathway diversity associated with polyketide and nonribosomal peptide biosynthesis were found in the 75 analyzed *Salinispora* genome sequences (Ziemert et al., 2014). Although 15 out of 30 strains were identical in both studies (Ziemert et al., 2014 and present study), we carried out this study not only in terms of the genomic but also the chemical perspective to identify genes and corresponding metabolites. Herein, we report a putative novel glycosylated NRPS/PKS-I hybrid metabolite and its gene cluster from *S. pacifica* CNY703 by using the MS-guided genome mining strategy. The combination of genomic and metabolomic data has been used to discover some novel compounds including anticancer retimycin A (Duncan et al., 2015), antifungal thanamycin (Kersten et al., 2011), and antibacterial vitroprocins produced by marine *Vibrio* sp. (Liaw et al., 2015). Kersten et al. (2013) established the connection between predictable glycosylation fragments from MS/MS experiments and underlined the glycosylation genes in microbial genomes. Cinerubin B is an exemplified compound that has been characterized as a glycosylated anthracycline antibiotic from a *Streptomyces* strain. Moreover, glycogenomics has facilitated the discovery of arenimycins, which are N-glycosylated aromatic polyketides from *S. arenicola* exhibiting significant anti-MRSA activity (Kersten et al., 2013).

### 3.3. Conclusions

In this study, we adopted genomics and metabolomics tools in order to highlight the metabolic potential of 30 marine obligate *Salinispora* strains. Fifteen strains of 30 were investigated for the first time with the goal of identifying genes and corresponding metabolites. Genome analyses of these strains by using two different genome mining software programs have provided comparisons of the natural product biosynthetic potential of *Salinispora* strains. The most important finding of this chemoinformatic study is to reveal the previously unknown NRPS/PKS-I gene cluster and unique signals belonging to its product in *S. pacifica* CNY703. Further studies are required to purify the compound and elucidate its structure.

## Acknowledgments

We are grateful to Dr Paul Jensen from the Scripps Institution of Oceanography, Center for Marine Biotechnology and Biomedicine (San Diego, CA, USA) for his guidance and insight throughout this research. This work was supported by a fellowship awarded to Süleyman Özakın by the Scientific and Technological Research Council of Turkey (TÜBİTAK 2214/A Fellowship Program).
